# An assessment of cetacean welfare in the Faroe Islands’ drive hunt

**DOI:** 10.1098/rsbl.2025.0311

**Published:** 2025-11-05

**Authors:** Alick G Simmons, Rebecca M. Boys, Laetitia Nunny, Mark P. Simmonds

**Affiliations:** ^1^ Independent researcher, Ilminster, UK; ^2^ School of Natural Sciences, Massey University Auckland, Auckland, New Zealand; ^3^ OceanCare, Gerbestrasse 6, Wädenswil 8820, Switzerland; ^4^ Bristol Veterinary School, University of Bristol, Bristol, UK

**Keywords:** animal welfare science, delphinid, Five Domains Model, killing, marine mammal, whaling

## Abstract

Drive hunts in the Faroes and Japan typically involve prolonged herding of cetaceans (over hours or even days) into shallow water, forced stranding, restraint and killing using a spinal lance and exsanguination. These methods raise significant animal welfare concerns. This study applies the Five Domains Model to the Faroe Islands’ *grindadráp* hunt to consider potential welfare implications and associated affective states. We examined published hunting guidelines and divided the hunt into six stages. Each author independently summarized potential impacts within the Five Domains Model; subsequent group consensus via online meetings assigned likely welfare impacts and affective states. Demonstrable welfare impacts and negative affective states were identified for each *grindadráp* stage. The prolonged chase, forced stranding, capture and restraint probably cause chronic and acute physiological stress. The spinal lance may not render animals instantaneously unconscious, raising substantial concerns that some animals may remain aware during exsanguination. Commonly inferred affective states for each domain included: pain, anxiety, disorientation, fear and panic. Given the inherent constraints of the hunt, it is unlikely that *grindadráp* can be undertaken humanely. If the hunt is to continue, substantial reform would be necessary to minimize animal suffering and align with welfare standards applicable to mammals in food production or research.

## 1. Introduction

Cetaceans are sentient animals [1–3], meaning that they have the capacity to consciously perceive by their senses, i.e. to consciously feel or experience subjectively [4]. Thus, concerns for cetacean welfare increasingly underpin policies around human interactions with them, both in captive and free-ranging settings (Article 13: [5, p. 13, 6,7]).

The capture and killing of small cetaceans in drive hunts, while once widespread, is now largely confined to the Faroe Islands in the northeast Atlantic and Japan [8]. During the ‘drive hunts‘ practised in these countries various species of small cetaceans are driven into shallow water to be killed for food and, in Japan, also for the acquisition of live dolphins for marine parks and aquaria [9]. Drive hunting methods are considered traditional by some [10], but are the subject of much controversy [9,11,12].

Previous research on cetacean drive hunts [9,13–15] suggests that there is potential for significant animal welfare impacts from the initial drive—which may result in avoidance behaviours and acute injury [9]—through the killing process, which is unlikely to cause a rapid death [14,16].

In the Faroe Islands, when the hunt focuses on long-finned pilot whales (*Globicephala melas*; which form the majority of cases), it is known locally as *grindadráp. Grindadráp* and its impact on animal welfare has been the subject of several studies [11,13,15,17]. The hunt is formed of three main parts with various stages in each: (i) the drive; (ii) the capture; and (iii) the killing of the animals:

(i) a pod of pilot whales or other dolphins is driven into shallow water usually in one of the several approved killing bays. In some cases, animals may be forced to strand on the beach and/or rocks;
(ii) the animals are hauled further into the shallows using a rope attached to a blunt hook which is placed in the blowhole until they are firmly secured. Simmons [15] concluded ‘that the drive hunt [...] falls short of the accepted handling standards used for other mammalian species, free-living or captive’ and that ‘the combination of noise and disorientation, the unfamiliar environment, the breaking-up of social groupings and the chase and capture are likely to cause considerable stress’; and
(iii) once restrained, the aim is to kill the animals using a bladed implement known as a spinal lance [18]. Butterworth [13] and Simmons [15] cast doubt on the assertion by the North Atlantic Marine Mammal Commission (NAMMCO; the body that advises governments on responsible hunting methods of marine mammals) that the spinal lance consistently both severs the spinal cord and destroys the arterial supply to the brain so that the animal ‘becomes unconscious and dies instantly’ [18]. While the effect of the spinal lance on the arterial supply to the brain has not been fully established—primarily owing to limited detailed understanding of the circulatory anatomy and dynamic physiology of the head and neck of pilot whales [19]—there is sufficient evidence from studies of mammalian physiology and the anatomy of cetacean arterial circulation to question whether the NAMMCO statement about unconsciousness and death being instantaneous can be supported [20, 14,19].

The NAMMCO instruction manual on pilot whaling (hereafter referred to as the NAMMCO manual) states that ‘the overriding principle pertaining to any killing is that it is carried out as quickly and painlessly as possible …’ [18], suggesting that the animal should be killed humanely. To ensure that any animal killing process is humane, a scientific assessment of animal welfare throughout the interaction (i.e. from initial drive and capture or transport to death) should be undertaken, and knowledge gained should be applied to improve animal welfare where possible [21,22].

While the Faroe Islands government and NAMMCO have striven to modernize *grindadráp* [18,23,24], there are still significant concerns about how the hunt impacts animal welfare [11,13,15]. The drive is one of the few events where wild mammals are driven in large numbers out of their natural environment and compares unfavourably with the pre-slaughter handling of terrestrial mammals, domestic or free-living [20,22,25,26].

Contemporary animal welfare science undertakes assessments based on the scientific understanding that the concepts of animal health, physiology, behaviour and affective experience or mental state are interrelated [27,28]. The Five Domains Model [29] is a welfare assessment framework that is widely used to systematically facilitate consideration of impacts in three physical/functional domains (nutrition, physical environment, and health; domains 1−3), the behavioural interactions that the animals may have with their environment, other non-human animals and with humans (behavioural interactions; domain 4) and the inferred impacts that these conditions have on the animal’s affective state (mental state; domain 5). It is the animal’s experience in the fifth domain that provides an understanding of its subjective welfare state. The model has been widely used for farmed, zoo and research animals [30–32].

Despite the growing acceptance of sentience in vertebrate animals and the understanding of human impacts on wildlife, assessment of animal welfare in free-ranging wild animals remains limited. However, those studies that have undertaken wild animal welfare assessments show that the Five Domains Model can be robustly applied [33–36], including to wild cetaceans [37–41].

The aim of this article is to highlight the potential welfare implications of *grindadráp*. We use the dominant Western animal welfare science perspective to contemporary animal welfare which emphasizes the acute mental experiences of the animal itself [29,42]. Specifically, we apply the Five Domains Model to systematically facilitate consideration of potential welfare impacts during each stage of the hunt and the probable associated subjective affective states.

### (a) Assessment of pilot whale welfare during *grindadráp*

Owing to the difficulties in observing the *grindadráp* directly [43], we considered the current version of the NAMMCO manual [18] to represent the most accurate description of the current processes involved in the hunt. We examined this published documentation that guides the hunt and dictates the procedures that should be undertaken [18] and a description of the development of the killing methods [23], to discern what each stage of *grindadráp* comprises. We also used detailed written descriptions and analyses of the hunt that have previously been published [11,13,15,44,45] to guide our assessment of the potential welfare impacts. Finally, we observed video footage that is publicly available to visually understand the different stages of the hunt (electronic supplementary material). This video footage was not analysed in this study as it did not focus on an individual animal throughout all stages of the hunt, and was used solely to inform the characterization of each stage as described in the NAMMCO manual [18].

**Figure 1.**
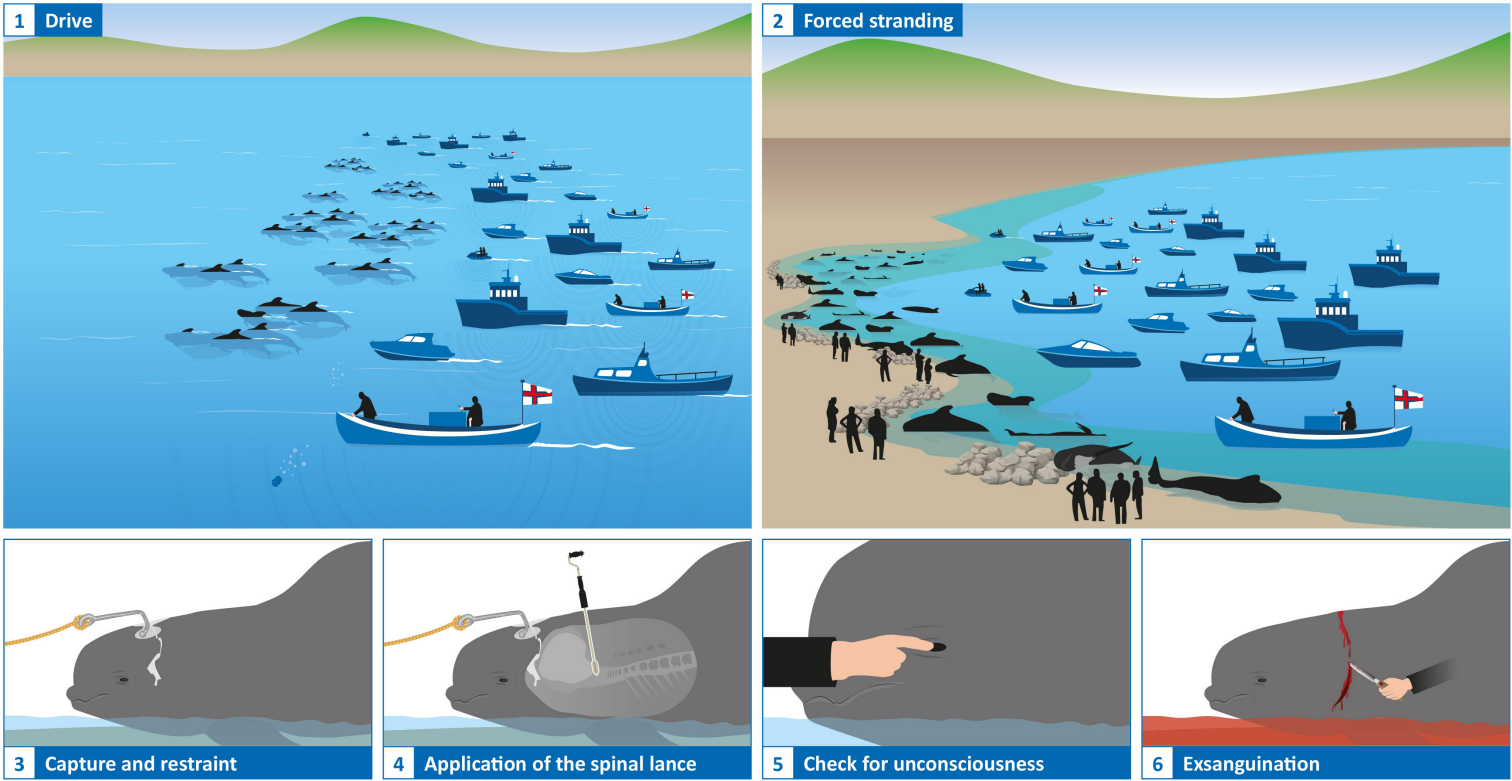
The six stages of *grindadráp* (images designed by the authors). See the electronic supplementary material for images taken from publicly available videos that show these stages from various *grindadráp* events.

Following examination of the documentation, we divided *grindadráp* into six stages: drive, forced stranding, capture and restraint, application of the spinal lance, check for unconsciousness, and exsanguination (figure 1). For each of the stages, the process was described and the advice and instruction from the NAMMCO manual [18] was quoted (table 1). Using the scientific published literature, we provided a summary of the potential impacts in each of the four physical/functional and behavioural domains of the Five Domains Model for each stage of *grindadráp* (table 2). Specifically, each of the authors individually placed potential impacts and possible associated affective states into the Five Domains Model. The authors then had several online meetings to discuss their individual conclusions and reached a group consensus for each of the domains. We used these final impacts and associated affective states to infer the potential cumulative impacts on the fifth domain to determine the overall potential subjective experiences and welfare state [14,39,78,79].

**Table 1.** Description of the six stages of *grindadráp* including instructions from the NAMMCO manual [18]. (See the electronic supplementary material for images taken from publicly available videos that show these stages from various *grindadráp* events.)

stage	description	instruction from NAMMCO manual [18]	NAMMCO statement relevant to potential welfare impact
1: drive	pilot whales or dolphins are driven into shallow bay using motor boats and noise. Although the objective is to capture and kill the entire pod, social groups within the pod are often broken up during the drive	‘When a school of pilot whales is sighted (either from land, sea or air), the district administrator, the foremen or both have to decide into which whaling bay the school shall be driven. Once the decision on location is made, the boats form in a semi-circle behind the whales under the direction of the foremen. Stones are thrown into the water to make air bubbles, which help herd the whales in the desired direction. Upon approaching the whaling bay, the boats are arranged by size, the smallest boats which can get closest to the beach, are in the front row, while the larger boats are kept behind’ (p. 11)	no claim
2: forced stranding	pilot whales or dolphins are forced to strand on beach/rocks, but usually to some extent in the water. Animals may attempt to right themselves to get their blowholes above the water surface, some may remain on their sides or upside down	‘The school is beached or driven so close to the beach that people are able to wade out to the whales to secure them for the killing’ (p. 11)	no claim
3: capture and restraint	blunt hook (with rope attached) inserted into blowhole. Individual cetaceans are dragged one by one into shallow water and onto beach where they are restrained	‘The blowhole hook is not inserted into the nostrils but in one of the two pocket-like formations, vestibular air sacs, which are located on either side of the blowhole between the skull and the skin […]. Although the surrounding tissue is solid and will withstand considerable strain, the use of the blowhole hook should be kept to a minimum’ (p 13). ‘The hunter securing the whale with the blowhole hook must stay with the whale as it is hauled up and until it is killed’ (p. 9)	no claim
4: application of the spinal lance	spinal lance is used to sever the spinal cord and associated blood vessels	‘The hook must remain in the blowhole and the air sacs during the killing and should not be pulled out before it has been confirmed that the whale is dead’ (p. 15). ‘The blade of the spinal lance must be perpendicular to the line between the blowhole and dorsal fin. The stab must be made perpendicular relative to the surface or be directed at a backward angle of approximately 10 degrees. Immediately after the severing of the spinal cord the lance must be moved to both sides in order to ensure that the surrounding blood vessels in the spinal canal are cut’ (p. 21)	‘When the spinal cut is performed […] with the spinal lance, the spinal cord is cut and the whale is paralysed and lies completely still. At the same time the blood supply to the brain is disconnected and the whale becomes unconscious and dies instantly’ (pp. 16 and 17)
5: check for unconsciousness	checking of the corneal reflex by touching the eye to assess if the animal is unconscious	‘After the movement associated with the cutting of the spinal cord and the severing of the spinal arteries, the whale will not move. The whale does not lose consciousness and die from the cutting of the spinal cord but by the successive cutting of the spinal arteries. The unconsciousness is confirmed by checking if the animal blinks (the corneal reflex) or the eye moves when touched. If there is no reaction the whale is unconscious or dead. The hunter using the spinal lance is obliged to do this check’ (p. 23)	‘If the whale blinks or moves the eyes it is regarded as conscious and needs to be re-stabbed’ (p. 21)
6: exsanguination	the animal is exsanguinated by cutting deep into neck—nominally after death	‘The whale bleeds extensively and dies after the cutting of the spinal arteries. However, in order to ensure good meat quality it is important to also cut the blood vessels at the throat after the whale is dead. This is best done by cutting deep into both sides of the neck and into the blood vessels with a whaling knife’ (p. 23)	‘The act of bleeding a whale must not commence before the hunter using the spinal lance has given his approval’ (p. 23)

**Table 2.** Potential impacts of *grindadráp* hunt organized into the three physical/functional domains and one situation-related domain, and the potential associated affective states experienced in the fifth domain of the Five Domains Model for welfare assessment [29]. (Impacts in domains 1−4 are stated at the expected first occurrence. Those in bold are likely to be ongoing throughout the subsequent stages of the hunt until the animal is irreversibly unconscious.)

stage (for stages 1, 5 and 6, two outcomes are possible: both are given here)		the Five Domains Model					supporting evidence
		domain 1: nutrition	domain 2: physical environment	domain 3: health	domain 4: behavioural interactions	domain 5: mental state	
**1: drive**	if stages 2−6 are carried out directly after the drive	— limited impact	unpredictable environment including: — vessels — loud noises — stones being thrown	— potential for wounding/injury from vessel strike and/or stones — sustained physiological stress response: — increased respiration — increased heart rate — release of significant amounts of catecholamines leading to, e.g. cardiomyopathy, rhabdomyolysis, myoglobinuric nephrosis	— interference with the expression of normal individual and group behaviour — **interference with group communication and physical interaction** — inability to comprehend and interact with environment	— anxiety — confusion — disorientation — fear — pain — panic	[9,18,39,46–61]
	if cetaceans are kept in the bay overnight before stages 2−6	— inability to feed — dehydration					
						— hunger — thirst possible owing to lack of foraging — weakness	
**2: forced stranding**			— **not fully supported by water causing unnatural pressure on organs**	— **respiratory difficulty** — inhalation of water if blowhole underwater — **incidental wounding (cuts/abrasions**) — **internal organ damage**	— **abnormal exertion** — **inability to move away from humans** — **lack of autonomy in body posture/movement**	— anxiety — breathlessness — confusion — discomfort — fatigue — fear — pain — panic	[13,39,45,62–68]
**3: capture and restraint**			— **skin desiccation and blistering**	— **trauma to blowhole** — **impairment of respiratory function because of positioning of blowhole hook** — **impaired circulation, hyperthermia and dehydration** — **further muscular myopathy owing to being restrained, i.e. capture myopathy**	— **inability to close blowhole** — **interference with the expression of normal stranding-related behaviour**	— anxiety — breathlessness — confusion — fear — helplessness — pain — panic — thermal discomfort — thirst — weakness	[18,39,65,69–74]
**4: application of the spinal lance**				– functional impairment owng to spinal cord being severed - blood and fluid loss leading to hypovolemic shock	— **inability to move (paralysis**) — **awareness of other animals being captured, restrained, lanced and exsanguinated**	— anxiety — dizziness — fear — helplessness — pain — panic — weakness	[ 20,64,72,75]
**5: check for unconsciousness**	if unconscious	no impact	no impact	no impact	no impact		
	if conscious			— temporary vision impairment	— human interaction with eye	— discomfort — irritation — fear — pain	[13,76,77]
**6: exsanguination**	if unconscious	no impact	no impact	no impact	no impact		
	if conscious					— anxiety — dizziness — fear — pain — weakness	[13,20,76]

## 2. Results and discussion

Table 2 shows the potential impacts in each of the domains for each stage of *grindadráp*. We found that for each stage of *grindadráp* there would be demonstrable impacts in three of the domains (physical environment, health, and behavioural interactions), all of which would have cumulative implications on overall animal welfare state. In the fifth domain, we inferred that one or more of the following negatively valenced affective states would be experienced for a given stage of *grindadráp*: anxiety, confusion, disorientation, dizziness, fear, panic, pain, discomfort and weakness.

We have assumed that the length of time between the beginning of the drive and the death of the animals is usually sufficiently short (based on the NAMMCO manual) that cetacean nutrition is unlikely to be compromised. However, the processes may go on for hours [11] and in some cases for several days [10]. For example, if the animals swim back out to sea rather than ashore or, during the short days of winter, animals may be confined alive overnight in a bay prior to being killed [52]. In these circumstances, the length of herding and confinement, and the probable inappropriate habitat for foraging are expected to negatively impact the nutrition domain. This could lead to animals experiencing welfare states of hunger, thirst and weakness [39,58].

In *grindadráp,* cetaceans are driven through a prolonged chase, potentially over several days [10]. This driving probably results in both acute and chronic physiological stress responses with significant welfare concerns (table 2). Indeed, cetaceans which undergo a sustained stress response will release significant amounts of catecholamines which may lead to capture myopathy and cardiomyopathy [9,50,55] impacts that are suggested to be painful [78]. Additionally, pilot whales are gregarious cetaceans living in close, stable groups, with long periods of maternal dependency [48,80]. Therefore, disruption to their social grouping through the chase, herding and separation, which occurs during hunts, is likely to negatively impact their welfare [9].

Cetaceans, unlike terrestrial mammals, do not have a skeleton capable of supporting their weight on land. When stranded, either in shallow water or on the beach, the internal organs become compressed [72,81], which can lead to pulmonary lesions and congestion [67], causing respiratory compromise and the negative welfare experience of breathlessness [39,62,78]. The longer the time between forced stranding and unconsciousness, the greater the welfare impact.

Compression of the cetacean body can also lead to ischaemia and reperfusion injuries [57,61] as well as rhabdomyolysis of skeletal and cardiac muscles [55,57,82], all of which are likely to cause pain [78]. Hyperthermia commonly occurs in stranded cetaceans owing to their compromised thermoregulatory ability out of water [72], and is indicative of welfare compromise experienced as the thermal discomfort of overheating. Additionally, their skin is not adapted to be out of water, rapidly becoming desiccated and sunburnt [71,83]. This will probably be experienced as the negative welfare states of pain and discomfort [83]. Such conditions are worsened during direct exposure to sunlight and warmer environmental conditions [65], and may lead to hypovolemic shock and dehydration [71,72].

Handling subsequent to forced stranding will also impact the welfare of captured cetaceans. For example, the use of hooks in highly innervated tissues around the blowhole [69,70] is likely to cause pain, and has the potential to hinder respiratory-related roles, such as complete closure of the blowhole to limit water ingress; human obstruction of such a survival-critical function would impact animal welfare, probably causing multiple forms of distress [29].

The subsequent killing is no less of a concern. The authorities advocate that killing should proceed rapidly [18,23], but Butterworth [13] examined video footage which showed that the period between forced stranding and killing is often 10 min or more. NAMMCO asserts that the spinal lance is an effective and humane means of killing pilot whales [18]. For this to be true, the animal would need to be rendered instantaneously and permanently unconscious, before or simultaneously with becoming paralysed by severing the spinal cord. For an animal to become instantly and irreversibly unconscious, the brainstem, which is key to the generation of conscious awareness through the reticular activating system [84,85], must be disrupted. However, there is no evidence that the brainstem and/or sufficient blood supply to the brain is consistently destroyed using the spinal lance [13,15].

There is evidence that cetacean anatomical and physiological adaptations enable the brain to function at relatively low arterial oxygen partial pressure (pO_2_) while diving, suggesting that cetaceans may be able to maintain consciousness when blood supply to the brain is interrupted. Although associated spinal blood vessels will be cut during the application of the spinal lance, it is possible that disruption to the cervical arterial system is incomplete and that an unknown amount of arterial, oxygenated blood continues to reach the brain. The proportion of the arterial supply to the brain that is closely associated with the spinal cord remains unknown, with some elements of the *retia mirabilia* lying ventral to the spinal column [86–88]. Since the path of the spinal lance as shown in the NAMMCO manual [18] does not appear to pass ventral to the spinal column, some blood supply may continue to the brain, probably enabling survival-critical cardiorespiratory systems and consciousness to function.

Unconsciousness must be confirmed following the application of any killing method to ensure a humane death. In mammals, including cetaceans, the most common validated criterion for verifying unconsciousness is the absence of the corneal reflex [21,38,76]. Indeed, the NAMMCO manual mandates that those applying the spinal lance confirm unconsciousness ‘by checking if the animal blinks (corneal reflex) or the eye moves when touched’ [18]. Despite the obligation to undertake this check, there is evidence that it is not routinely carried out [13], highlighting the potential for continued animal consciousness and awareness while paralysed, leading to significant welfare compromise during the killing process. A similar concern has been raised about drive hunts in Japan, where severing of the spinal cord is also applied [14,89].

Furthermore, the NAMMCO manual suggests that ‘the whale […] dies instantly’ following the application of the spinal lance [18]. However, animal death will only occur once the circulatory system stops functioning, i.e. heartbeat ceases. Respiration and modulation of the heartbeat will cease if the brainstem is sufficiently disrupted, since this brain region is responsible for cardiorespiratory function [87,90,91]. However, the heart will continue to beat independently until spinal-associated and cervicothoracic blood vessels are cut and oxygen supply to cardiac muscles is no longer available, i.e. exsanguination. Although substantial haemorrhaging will occur during spinal transection, as the rete and vertebral blood vessels are disrupted, research suggests that this will not lead to a rapid death in such large mammals [14,20]. Therefore, the animals will not ‘die instantly’ after application of the spinal lance; indeed, research on stranded cetaceans euthanized via physical disruption of the brain has found that a heartbeat can still be evident minutes after unconsciousness has been verified [79].

NAMMCO states that a whaling knife (in Faroese *grindaknívur*), which usually has a blade between 16 and 19 cm, should be used to sever the remaining ‘blood vessels at the throat after the whale is dead’ [18]. Given the evidence that unconsciousness is not routinely assessed [13], such exsanguination procedures are potentially being applied to conscious animals. This would cause significant welfare compromise and would not meet the requirements of a humane death (‘quickly and painlessly’*)* as mandated by NAMMCO [18]. Faroese animal welfare law requires the handling and killing of animals to be carried out humanely [92]. This paper and previous research on drive hunts [15,89,93] bring into question whether *grindadráp* complies with the requirements stipulated within the law.

## 3. Conclusion

By systematically considering the impacts of *grindadráp* through the Five Domains Model, we have provided an indication of the welfare impacts on cetaceans at each stage of the process. The prolonged driving, subsequent forced stranding and capture and restraint of the animals will severely compromise welfare, probably causing substantial pain and distress. The application of the spinal lance is unlikely to render animals instantaneously unconscious, and the lack of verification of unconsciousness suggests that at least some animals will probably be exsanguinated while remaining aware but paralysed. This will lead to significant welfare compromise prior to blood loss causing irreversible unconsciousness [76]. Such a method of killing is typically not considered humane nor legal in the slaughter of other mammal species used for human consumption (e.g. farmed animals) or for mammals used in research [16,94], though there are exceptions.

Given the inherent constraints of the hunt, particularly the means of capture and killing, we believe it is unlikely that *grindadráp* can be undertaken humanely. If the hunt is to continue and animal welfare is to be protected, radical change is necessary. We encourage the relevant authorities in the Faroe Islands to fully consider the welfare implications of *all* stages of the drive hunt in light of the concerns highlighted in this paper.

## Data Availability

Our study draws on published information in the form of referenced scientific papers. The authors did not draw on an existing dataset nor collect new data. Supplementary material is available online [95].
